# Study of females of *Podocnemis unifilis *(Tracajá) (Troschel, 1848) in areas between Xambioá-TO, Antonina-PA and Tucuruí-PA, by molecular tools

**DOI:** 10.1186/1753-6561-8-S4-P250

**Published:** 2014-10-01

**Authors:** Maria Augusta Agostini, Deyla Paula Oliveira, Izeni P Farias, Waldesse Piragé Oliveira Junior

**Affiliations:** 1 Federal University of Tocantins, Tocantins, Brazil; 2 Federal University of Amazonas, Manaus, AM, Brazil

## Background

The turtles are usually associated with low substitution of individuals in the population because they have longer life than any other vertebrates. The order Testudines or Chelonia, the oldest among all vertebrates, comprising the turtles, terrestrial, marine and freshwater. These have as main characteristics, low growth rates and long periods required for mature, causing many turtles are vulnerable to extinction. Like other turtles, *P. unifilis*, popularly known as tracajá, leaves long-lived animals with late sexual maturity, characterized by a small mortality of adult animals, but high mortality rate of embryos and hatchlings, with natural predation as one of the most important factors of low hatching success of these species. This species is one of the most important representatives of the fauna of the Amazon turtle, but their eggs, meat and offal provide food for local communities, and have their hooves used as adornment and household items. The implementation of large hydropower enterprise cause many environmental damage. The scientific emphasis in EIA/RIMA should be held primarily in the study of special targets, and should take into account the genetic structure of populations. Mitochondrial molecular markers have particular maternal characteristics and high rates variation, which allow us to analyze the behavior of populations. The deployment of hydropower modifies the natural habitat of the turtles, which have difficulty in adapting. This work aimed to study the genetic structure of populations of *P. unifilis *analyzing the behavior of females in conditions of ambient modified by Tucuruí, and Antonina-PA and Xambioá-TO, where will be built the dam of Santa Isabel.

## Methods

We performed the sequencing of fragments of the control region of mitochondrial DNA, generating data of gene diversity and nucleotide, numbers of haplotypes and segregating sites, AMOVA, levels of structuring by pairwise comparison of ΦST and Mantel test in ARLEQUIN program. Clades were grouped checked by software Treefinder and the haplotype tree was held at hapview software.

## Results and conclusions

The study populations were similar to each other and have high diversity within them, but the region of rocks and waterfall in Xambioá associated with hydroelectric Tucuruí, act as barriers to gene flow. Tucuruí has the largest number of groups haplotypes, upriver this number decreases by Antonina and especially Xambioá. The Mantel test showed no correlation between genetic variation and spatial distribution, noting that the species does not present a long-distance migratory pattern. The region Xambioá and Tucuruí should be considered distinct management units due to the influence of anthropogenic activities differently in each place.

## References

[B1] Alho CJR Pádua LFM Reproductive parameters and nesting behavior of the Amazon turtle Podocnemis expansa (Testudinata: Pelomedusidae) in Brazil Canadian J Zool 1982 60 97 103 10.1139/z82-012

[B2] Fachín-Terán A Vogt RC Estrutura populacional, tamanho e razão sexual de Podocnemis unifilis (Testudines, Podocnemididae) no rio Guaporé (RO), Norte do Brasil Phyllomedusa 2004 3 1 29 42 10.11606/issn.2316-9079.v3i1p29-42

[B3] Mantel N The detection of disease clustering and a generalized regression approach CancerResearch 1967 27 209 220 6018555

[B4] Pritchard PCH Trebbau P The Turtles of Venezuela Society for the Study of Amphibians and Reptiles 1984 403

[B5] Valle RC Alfinito J Silv AMMF Contribuição ao estudo da tartaruga amazônica. Preservação da tartaruga da Amazônia Ministério da Agricultura 1973 DEMA/ P A, IBDF, Belém (P A) 66 88

